# Oxytocin Response Following Playful Mother–Child Interaction in Survivors of the Great East Japan Earthquake

**DOI:** 10.3389/fpsyt.2020.00477

**Published:** 2020-06-03

**Authors:** Nobutoshi Nawa, Kazuaki Nakamura, Takeo Fujiwara

**Affiliations:** ^1^Department of Global Health Promotion, Tokyo Medical and Dental University, Tokyo, Japan; ^2^Institute of Education, Tokyo Medical and Dental University, Tokyo, Japan; ^3^Department of Medical Education Research and Development, Tokyo Medical and Dental University, Tokyo, Japan; ^4^Department of Pharmacology, National Research Institute for Child Health and Development, Tokyo, Japan; ^5^Department of Social Medicine, National Research Institute for Child Health and Development, Tokyo, Japan

**Keywords:** oxytocin, the Great East Japan Earthquake, parenting, child behavior problems, heart rate variability

## Abstract

**Background:**

Children who are exposed to natural disasters are at greater risk of developing mental and behavior problems. Prior studies have suggested that positive parenting practices could prevent child mental and behavior problems among those who were exposed to natural disasters. Parent–child interaction increases oxytocin level in parents and infants; however, studies assessing the change in oxytocin level after positive parent-child interaction and its effect on child behavior problems among preadolescents who were exposed to natural disasters are lacking. This study investigated whether playful interaction stimulated oxytocin levels in 34 mother–child dyads who experienced the 2011 Great East Japan Earthquake in Kesennuma City in Miyagi Prefecture, Japan, and the effect of the maternal oxytocin changes on child behavior problems.

**Methods:**

Participants were recruited in 2012 after the Great East Japan Earthquake. Annual surveys were conducted from 2012 to 2017. Salivary oxytocin level was assessed before and after the playful interaction in 2015. Behavior problems were evaluated by caregivers, using the Child Behavior Checklist (CBCL) in 2017. Fixed effect regression analyses were conducted to determine the effect of playful mother–child interaction on oxytocin level by comparing the change in the 10 min after the interaction with the change in the 10 min before the interaction. We also examined the effect of maternal oxytocin changes before and after the playful interaction on the onset of child behavior problems in 2017.

**Results:**

A significant increase in maternal oxytocin level was detected following playful interaction, especially among mothers of first-born boys (2.63 pg/mg protein. 95% CI: 0.45, 4.81). Maternal psychological distress and trauma were also negatively associated with an increase of oxytocin levels. The increase in maternal oxytocin level was significantly associated with lower externalizing problem score of children 2 years later.

**Conclusion:**

Our results might suggest a rational for potential preventive intervention for child behavior problems through playful mother–child interaction after natural disasters.

## Introduction

Numerous studies have shown an increasing frequency and severity of natural disasters in many parts of the world (1–5). Children who are exposed to natural disasters are at greater risk of developing posttraumatic stress disorder (PTSD), depressive symptoms, anxiety, and behavior problems (6–10).

Previous studies have tested a wide variety of interventions with the goal of alleviating deleterious effects of disaster experiences on mental health, emotional well-being, and behavior in children (9, 11–13). These preventive measures include cognitive behavioral approaches, provision of social support, and development of coping skills. Many of the interventions are provided in schools by mental health professionals or school personnel (9, 11, 13). Notably, an association between parenting practices and favorable parent–child relationships and child mental symptoms after disaster exposure has been observed (7, 14, 15). These studies suggest programs that promote positive parenting practices prevent child mental and behavior problems among those who were exposed to natural disasters.

Prior studies reported that parenting behaviors were partly affected by oxytocin (16–18). Intranasal administration of synthetic oxytocin led to the induction of maternal behavior in rats (19) and increased gazing behavior in dogs (20). In humans, intranasal administration of oxytocin was associated with improvement in protective behavior towards their children in depressed mothers (16, 21). Feldman et al. reported that parental interaction with their infant by itself increased oxytocin level in parents and infants (17). Stimulating natural secretion of oxytocin by parent–child interaction would be better suited to large-scale parenting programs among disaster survivors, because it would be more accessible and economical than intranasal administration of oxytocin. However, studies assessing the change in oxytocin level after parent–child interaction among preadolescents who were exposed to natural disasters are lacking.

Moreover, to develop a practical intervention after natural disaster, heterogeneity of subjects needs to be considered. The effectiveness of parenting intervention may depend on factors such as specific trauma exposure and the birth order or sex of the child. Assessment of change in oxytocin level due to parenting among those who were exposed to natural disasters, stratified by trauma type, and the children's characteristics, may provide the rationale for parenting intervention after natural disasters.

The Great East Japan Earthquake and tsunami struck Japan on 11 March 2011, causing more than 15,000 deaths and damaging over 400,000 houses, together with the evacuation of nearly 470,000 people (22, 23). About 18 months after the earthquake, we recruited children aged 3–5 years old at the time of the earthquake to investigate the impact of the earthquake on young children (24). The objectives of the study were: 1) to examine whether experimental playful interaction stimulated oxytocin level in mothers and children who were exposed to the Great East Japan Earthquake in Kesennuma city in Miyagi, Japan, 2) to examine the effect of the interaction on short-term psychophysiological status using heart rate variability (HRV), and 3) to assess the effect of maternal oxytocin changes due to interaction on the onset of child behavior problems 2 years later.

## Methods

### Sample

Participants included 34 mother–child dyads from the cohort in Kesennuma, Miyagi, Japan, who were recruited using a multistage sampling method after the Great East Japan Earthquake in 2012. The detail of the cohort was described elsewhere (24). We selected Kesennuma city, because it was severely affected by the earthquake, the resulting tsunami, and fire. Preschools in the selected municipality were invited to participate, and two of 16 preschools agreed to participate. Mothers with children aged 3 to 5 years old were invited to join the study by the principals or staff of the preschools in 2012.

Because of the limited language skills of preschool children, the effect of exposure to trauma due to the earthquake is difficult to assess and has been understudied (25). We initiated this cohort in Kesennuma, Miyagi, to clarify the effect of exposure to trauma due to the earthquake in preschool children (24, 25). In our prior study, using data from this cohort, we reported an association between facial expressions of preschool children, measured by facial expression recognition software, and child PTSD symptoms (25).

We conducted annual surveys from 2012 to 2017. Enrolled mothers provided our research coordinators with written informed consent for their child, completed the questionnaire, and conducted playful mother–child interaction. Informed consent was also obtained to publish the image from the participant for **Figure 2**. After excluding one mother for incomplete oxytocin measurement and five children (three for incomplete oxytocin measurement, one for incorrect measurement, and one for missing identification), an analytic sample of 33 mothers and 29 children was obtained. This study was approved by the research ethics committees at the National Center for Child Health and Development (584) and the Ethics Committee at Tokyo Medical and Dental University (M2016-148). This study was performed in accordance with the Declaration of Helsinki and the Japan's Ethical Guidelines for Epidemiological Research established by the Ministry of Education, Culture, Sports, Science and Technology and the Ministry of Health, Labour, and Welfare.

### Procedure

**Figure 1:**
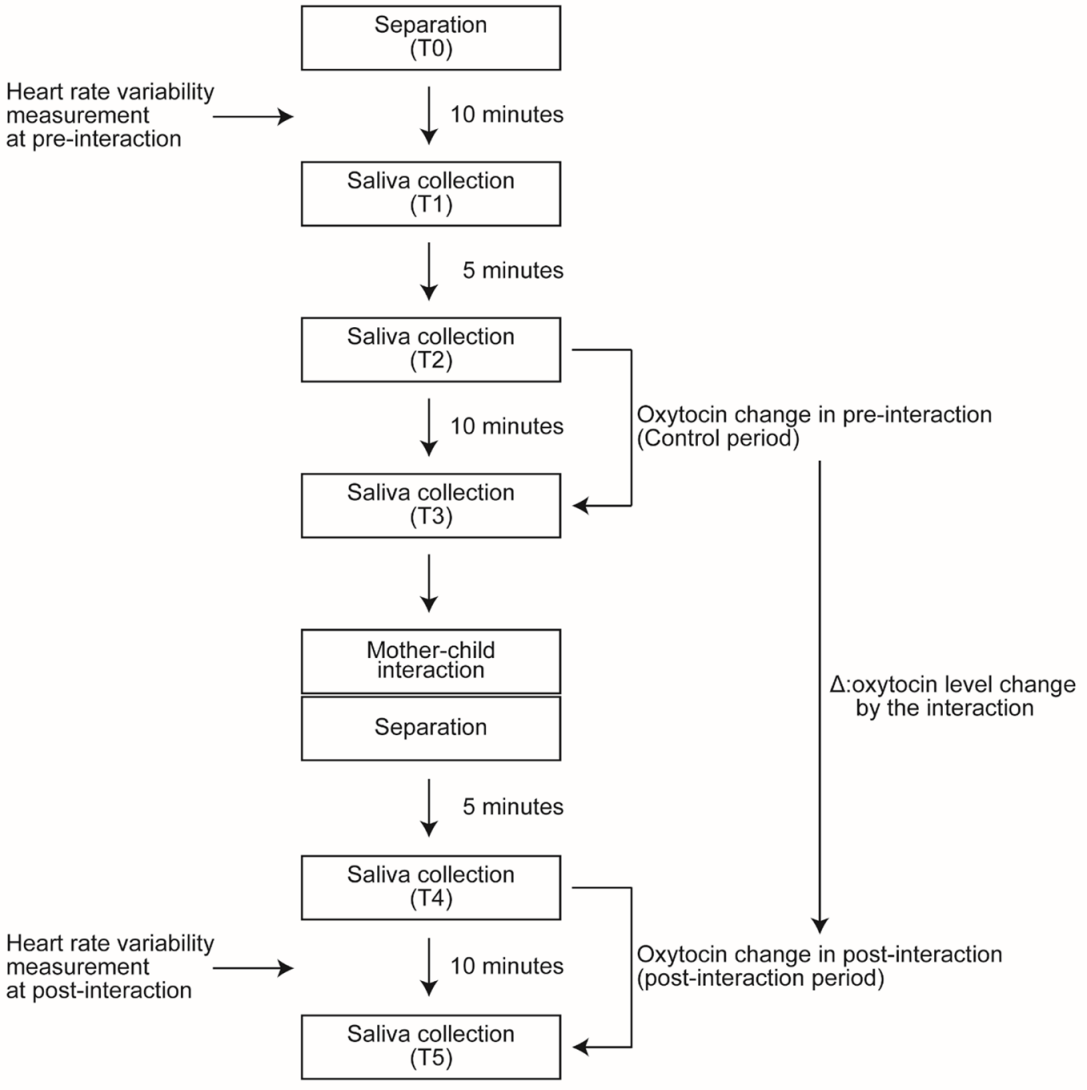
Study protocol. Study protocol from time point zero (T0) to time point five (T5).

In the annual survey conducted in 2015, as shown in **Figure 1**, after the participants arrived at the examination lab, the mother and children were asked to wait in separate rooms (Time point 0: T0). During the next 10 min, HRV of mothers and children in the pre-interaction period was measured. Ten minutes after T0, the first saliva sample collection was made (Time point 1: T1). During the pre-interaction period (control period), samples were collected twice: 5 min after T1 (Time point 2: T2) and 10 min after T2 (Time point 3: T3). Similarly, during the post-interaction period, sample were collected twice: 5 min after the interaction (Time point 4: T4) and 10 min after T4 (Time point 5: T5). During the 10 min between T4 and T5, HRV of mothers and children in the post-interaction period was measured. We assessed the effect of playful mother–child interaction on oxytocin level by comparing the change after the interaction (from T4 to T5 in **Figure 1**) with the change before the interaction (from T2 to T3 in **Figure 1**).

### Intervention: Playful Mother–Child Interaction (Conducted in 2015)

**Figure 2:**
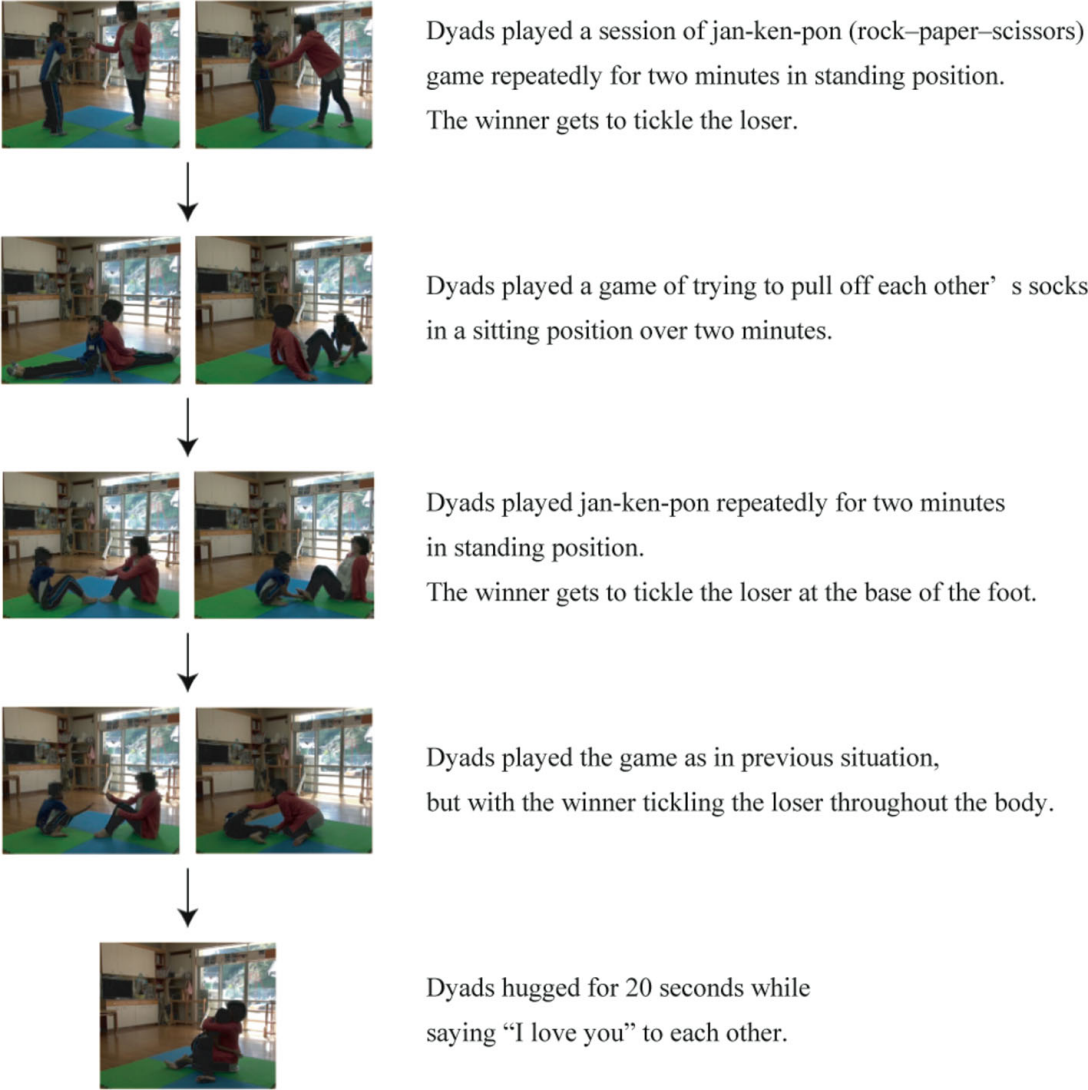
Intervention: Playful Mother–Child Interaction. Intervention protocol with representative images for each procedure was shown.

We developed the following procedure for playful mother–child interaction based on previous studies (26, 27) (**Figure 2**). The intervention was conducted on a 3.2 m^2^ mattress in a 50–100 m^2^ room in either a local health center or a nursery school in Kesennuma city. The entire intervention was video-taped for quality control.

1. In a standing position, the dyads conducted a session of *jan-ken-pon* (rock–paper–scissors), with the winner tickling the loser. The game was played repeatedly for 2 min.
2. In a sitting position, the dyads played a game that involved trying to pull off each other's socks for 2 min.
3. In a sitting position, the dyads played a session of *jan-ken-pon*, with the winner tickling the loser at the base of the foot. The game was played repeatedly for 2 min.
4. In a sitting position, the dyads played a session of *jan-ken-pon*, with the winner tickling the loser all over their body. The game was played repeatedly for 2 min.
5. Each of the dyads hugged for 20 s and told each other, “I love you”. The timing of the saliva sample collection is described in the following section for oxytocin measurement (**Figure 1**).

### Measures

#### Oxytocin

We collected five saliva samples from each dyad (**Figure 1**). Saliva samples were collected by Salivette (Sarstedt, Nümbrecht, Germany) and kept frozen at −80^◦^C until the assay. Salivary oxytocin level was measured using a commercial ELISA kit (Assay Designs, Ann Arbor, MI, USA) (28). The data were normalized by protein concentration in the saliva (pg/mg salivary protein).

#### Heart Rate Variability

In the 2015 annual survey, HRV was assessed at two time points (**Figure 1**), with a TAS9 pulse analyzer (YKC, Tokyo, Japan) by recording pulse rate for 5 min. In the time-domain, inter-beat intervals (R-R intervals, RRI) were calculated. Then, the standard deviation of normal to normal R-R intervals (SDNN) and the root mean square of successive differences (RMSSD) were assessed. For the analyses of HRV in the frequency-domain, the signal was filtered into different frequency bands: a low-frequency (LF) (0.04–0.15 Hz) and high-frequency component (HF) (0.15–0.40 Hz). The LF/HF ratio was used for further analyses (29–31). LF, HF, and LF/HF ratio were analyzed in the log scale.

#### Other Variables

Information on the following variables were assessed using questionnaires that were administered to mothers from September 2012 to June 2013: maternal age, child sex, birth order of the child, K6 (32, 33), Alabama Parenting Questionnaire (APQ) (34), and whether they experienced damage to their house, witnessed the tsunami, or heard the explosion of multiple oil tanks (32, 33). K6 is a six-item scale used for screening psychological distress and has a score ranging from 0 to 24 with a higher score indicating more frequent psychological distress (33). The Japanese version of K6 was used in this study (32, 33). For assessing maternal parenting, we used APQ (34), which has five subscales: involvement, positive parenting, poor monitoring, inconsistent care, and corporal punishment (35–37). The total APQ score was used for the analysis after dividing it into two categories at the median. Behavior problems were evaluated by caregivers, using the Child Behavior Checklist (CBCL) in 2017 (38). The T score of the CBCL internalizing, externalizing, and total problem scores were calculated using the standardized distribution of Japanese children (39).

### Statistical Analyses

In order to account for the within-individual association between repeatedly measured data from the same child, fixed effect regression analyses were conducted to determine the effect of playful mother–child interaction on oxytocin level by comparing the change after the interaction with the change before the interaction. Samples were also stratified by maternal and child characteristics, such as sex of the child, birth order of the child, K6, and whether they experienced damage to their house, witnessed the tsunami, or heard the explosion of multiple oil tanks, to examine whether the effects varied depending on maternal and child characteristics. Fixed effect regression analyses were also conducted to determine the effect of the playful mother–child interaction on HRV in mothers and children. We fit linear regression models to assess the association between maternal change in oxytocin level following playful mother–child interaction in 2015 and the CBCL score 2 years later, adjusting for mother's age, sex of the child, APQ, and child traumatic experiences due to the earthquake. All analyses were conducted using STATA SE statistical package, version 14 (StataCorp LP, College Station, TX, USA).

## Results

### Characteristics of the Sample Population

**Table 1** shows the characteristics of the sample population. The mean maternal age was 38.5 years (SD: 5.1). About 40%–60% of mothers experienced hardships caused by the earthquake such as damage to the house, experience of living in a shelter, witnessing the resulting destructive tsunami, and hearing the explosion of multiple oil tanks. About one-third of the mothers reported psychological distress as defined by K6 score of 5 or more (32, 33). The mean age of the children was 8.8 years (SD: 0.9), with two-thirds being female, and about half being a first-born child.

**Table 1:** Characteristics of sample population.

Variable		Total (n=33)	
		N or Mean	% or SD
Maternal characteristics			
Age in 2014	Mean (years old)	38.5	5.1
House damaged	Yes	16	48.5
	No	17	51.5
	Missing	0	0
Lived in a shelter	Yes	14	42.4
	No	17	51.5
	Missing	2	6.1
Witnessed tsunami	Yes	22	66.7
	No	10	30.3
	Missing	1	3.0
Heard the explosion of multiple oil tanks	Yes	16	48.5
	No	14	42.4
	Cannot answer	1	3.0
	Missing	2	6.1
K6	≤ 4	23	69.7
	≥ 5	9	27.3
	Missing	1	3
Child characteristics			
Age in 2014	Mean (years old)	8.8	0.9
Sex	Male	13	39.4
	Female	20	60.6
Birth order	1^st^	17	51.5
	≥ 2^nd^	14	42.4
	Missing	2	6.1

### Mean Oxytocin Levels of Mothers and Children at Each Time Point

**Table 2** shows the mean oxytocin levels of the mothers and children at each time point of the measurement. A negative change in maternal mean oxytocin level in the pre-interaction period was detected (from 10.14 to 9.31 pg/mg protein). A decreasing trend was observed in both the mothers with boys and mothers with girls. In the post-interaction period, a positive change in maternal mean oxytocin level was detected during the same 10 min under resting conditions (from 9.47 to 10.06 pg/mg protein). Although the increasing trend was observed in both the mothers with boys and mothers with girls, it was more evident in mothers with boys (mothers with boys: from 9.48 to 10.88 pg/mg protein; mothers with girls: from 9.46 to 9.53 pg/mg protein).

**Table 2:** Mean oxytocin level of mothers and children at each time point.

	Pre-experiment	Pre-interactionperiod (control period)		Change of pre-interaction period	Post-interaction Period		Change of post-interaction period	Change by playful interaction (interaction effect)	*t*	*P-value*
	T1	T2	T3		T4	T5				
**All Mothers**										
Mean	13.17	10.14	9.31	-0.83	9.47	10.06	0.60	**1.42**^**a**^	**2.34**^**a**^	**0.03**^**a**^
SD or SE	8.79	4.27	4.00	0.43^b^	4.93	4.82	0.46^b^	0.61^b^		
**Boy's mothers**										
Mean	11.75	9.95	8.72	-1.23	9.48	10.88	1.40	**2.63**^**a**^	**2.63**^**a**^	**0.02**^**a**^
SD or SE	6.65	4.28	3.15	0.78^b^	4.77	5.85	0.89^b^	1.00^b^		
**Girl's mothers**										
Mean	14.10	10.26	9.69	-0.56	9.46	9.53	0.07	0.63	0.87	0.40
SD or SE	10.00	4.36	4.50	0.50^b^	5.15	4.09	0.47^b^	0.73^b^		
**All Children**										
Mean	10.50	9.63	9.42	-0.22	9.55	8.83	-0.71	-0.50	-1.05	0.30
SD or SE	4.19	4.06	3.18	0.45^b^	3.45	2.29	0.45^b^	0.47^b^		
**Boys**										
Mean	10.52	10.10	10.70	0.60	9.53	9.94	0.41	-0.19	-0.32	0.76
SD or SE	2.86	2.30	2.64	0.48^b^	1.45	1.62	0.50^b^	0.60^b^		
**Girls**										
Mean	10.50	9.39	8.74	-0.64	9.56	8.25	-1.31	-0.66	-1.00	0.33
SD or SE	4.82	4.77	3.30	0.63^b^	4.18	2.41	0.60^b^	0.66^b^		

Saliva oxytocin values are in pg/mg protein. T, time point.

a P < 0.05 by paired t-test (two-sided).

b standard error was displayed.

In the children, a negative change in oxytocin level from T2 to T3 was detected (from 9.63 to 9.42 pg/mg protein). Similarly, a negative change in the child mean oxytocin level from T4 to T5 in the post-interaction period was detected (from 9.55 to 8.83 pg/mg protein).

### Maternal Oxytocin Response Following Mother–Child Interaction

**Figure 3:**
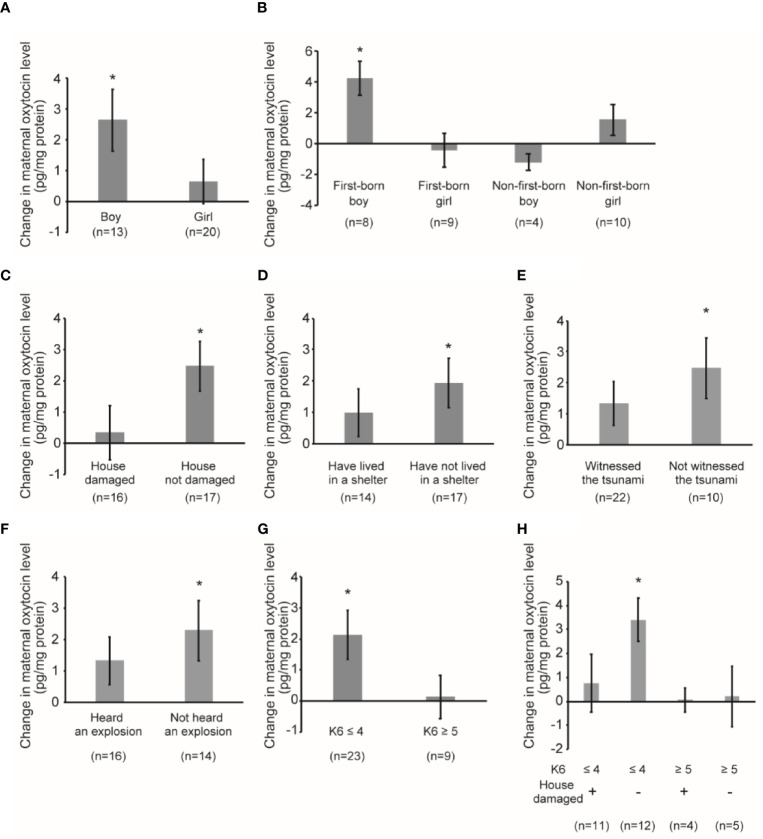
Maternal oxytocin response following playful mother–child interaction. **(A)** Change in maternal oxytocin levels by playful mother–child interaction by stratifying by child sex. *P < 0.05. **(B)** Change in maternal oxytocin levels by playful mother–child interaction by stratifying according to sex of the child as well as the birth order of the child. *P < 0.05. **(C)** Change in maternal oxytocin levels by playful mother–child interaction by stratifying by the presence of damage to the house by the earthquake. *P < 0.05. **(D)** Change in maternal oxytocin levels by playful mother–child interaction by stratifying by maternal experience of having lived in a shelter after the earthquake. *P < 0.05. **(E)** Change in maternal oxytocin levels by playful mother–child interaction by stratifying by the maternal experience of having witnessed tsunami after the earthquake. *P < 0.05. **(F)** Change in maternal oxytocin levels by playful mother–child interaction by stratifying by the maternal experience of having heard the explosion of multiple oil tanks after the earthquake. *P < 0.05. **(G)** Change in maternal oxytocin levels by playful mother–child interaction by stratifying by maternal psychological distress as assessed by K6 score (≤4 vs ≥5). *P < 0.05. **(H)** Change in maternal oxytocin levels by playful mother–child interaction by stratifying by maternal psychological distress as assessed by K6 score (≤4 vs ≥5) and the presence of damage to the house by the earthquake. *P < 0.05. All data and graph bars are expressed as the mean ± standard error. Saliva oxytocin values are in pg/mg protein.

**Figure 3** shows the maternal oxytocin responses due to playful mother–child interaction. An increase in maternal oxytocin level was detected among all mothers after the playful mother–child interaction of 1.42 pg/mg protein (95% CI 0.18, 2.66, *p*=0.026). In mothers with boys, we observed a significant increase in maternal oxytocin level of 2.63 pg/mg protein (95% CI 0.45, 4.81, *p*=0.022), whereas in mothers with girls, the change was not significant (coefficient 0.63 pg/mg protein, 95% CI -0.90, 2.17, *p*=0.397) (**Figure 3A**). The sample was stratified using the sex and birth order of the child (**Figure 3B**). In mothers with a male first-born, we detected a significant increase of 4.24 pg/mg protein (95% CI 1.66, 6.83, *p*=0.006); however, in mothers with a female first-born, the change was minimal and not significant (coefficient -0.42 pg/mg protein, 95% CI -2.99, 2.14, *p*=0.714). We did not find significant changes in mothers with a non-first-born boy or girl (non-first-born boy: coefficient -1.20 pg/mg protein, 95% CI -2.95, 0.54, *p*=0.116; non-first-born girl: coefficient 1.54 pg/mg protein, 95% CI -0.80, 3.87, *p*=0.170) (**Figure 3B**).

In mothers whose house was not damaged by the earthquake (**Figure 3C**), we observed a significant increase in maternal oxytocin level of 2.46 pg/mg protein (95% CI 0.76, 4.16, *p*=0.007). However, in mothers whose house was damaged by the earthquake, the increase was small and not significant (coefficient 0.32 pg/mg protein, 95% CI -1.52, 2.15, *p*=0.718). In mothers who lived in a shelter after the earthquake (**Figure 3D**), the change was smaller and not significant (coefficient 0.98 pg/mg protein, 95% CI -0.67, 2.63, *p*=0.221), whereas the change was larger and significant in those who did not live in a shelter (coefficient 1.92 pg/mg protein, 95% CI 0.25, 3.59, *p*=0.027). In mothers who witnessed the destructive tsunami after the earthquake (**Figure 3E**), the change was smaller and not significant (coefficient 1.32 pg/mg protein, 95% CI -0.12, 2.77, *p*=0.070), whereas the change was larger and significant in mothers who did not witness the tsunami (coefficient 2.47 pg/mg protein, 95% CI 0.26, 4.69, *p*=0.032). Similarly, in mothers who heard the explosion of multiple oil tanks (**Figure 3F**), the change was smaller and not significant (coefficient 1.31 pg/mg protein, 95% CI -0.29, 2.91, *p*=0.102), whereas the change was larger and significant in mothers who did not hear the explosion (coefficient 2.28 pg/mg protein, 95% CI 0.20, 4.35, *p*=0.034).

We also performed subgroup analysis stratified by maternal psychological distress as assessed by K6 score (**Figure 3G**). In mothers who did not report psychological distress, the change was larger and significant (coefficient 2.13 pg/mg protein, 95% CI 0.52, 3.74, *p*=0.012), whereas the change was minimal in mothers with psychological distress (coefficient 0.14 pg/mg protein, 95% CI -1.46, 1.75, *p*=0.843). We stratified using K6 and whether the respondent experienced damage to their house by the earthquake (**Figure 3H**). In mothers who did not report psychological distress and whose house was not damaged by the earthquake, the change was the largest and significant (coefficient 3.40 pg/mg protein, 95% CI 1.42, 5.38, *p*=0.003). In contrast, in mothers who did not report psychological distress and whose house was damaged by the earthquake, the change was smaller and not significant (coefficient 0.75 pg/mg protein, 95% CI -1.93, 3.43, *p*=0.547). In mothers with psychological distress, the change was minimal and not significant, irrespective of the damage to their house (**Figure 3H**).

### Child Oxytocin Response Following Mother–Child Interaction

**Figure 4:**
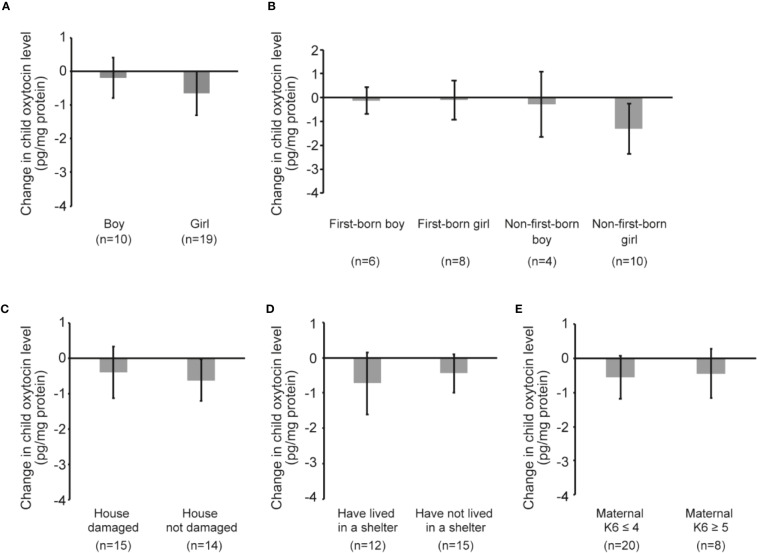
Child oxytocin response following playful mother–child interaction. **(A)** Change in child oxytocin levels by playful mother–child interaction by stratifying by sex of the child. **(B)** Change in child oxytocin levels by playful mother–child interaction by stratifying by sex of the child as well as the birth order of the child. **(C)** Change in child oxytocin levels by playful mother–child interaction by stratifying by the presence of damage to the house by the earthquake. **(D)** Change in child oxytocin levels by playful mother–child interaction by stratifying by the maternal experience of having lived in a shelter after the earthquake. **(E)** Change in child oxytocin levels by playful mother–child interaction by stratifying by maternal psychological distress as assessed by K6 score (≤4 vs ≥5). All data and graph bars are expressed as the mean ± standard error. Saliva oxytocin values are in pg/mg protein.

**Figure 4** shows child oxytocin response due to playful mother–child interaction. The change was small in both boys and girls (boys: coefficient -0.19 pg/mg protein, 95% CI -1.54, 1.16, *p*=0.757; girls: coefficient -0.66 pg/mg protein, 95% CI -2.04, 0.72, *p*=0.329) (**Figure 4A**). **Figure 4B** shows the results of the analyses with data stratified by the sex and birth order of the child. The changes were in the direction of a decreasing trend, although none of the changes were significant. **Figures 4C–E** show the oxytocin response following playful mother–child interaction according to maternal experience of the earthquake and maternal level of psychological distress. In **Figure 4C**, we observed some decreases in both children whose house was damaged and children whose house was not damaged by the earthquake, although the changes were not significant (damaged: coefficient -0.39 pg/mg protein, 95% CI -1.99, 1.21, *p*=0.608; not damaged: coefficient -0.61 pg/mg protein, 95% CI -1.90, 0.67, *p*=0.321). Similarly, **Figure 4D** shows that there were some decreases in both children of mothers who lived in a shelter and children of mothers who did not live in a shelter; however, the results were not significant (lived in a shelter: coefficient -0.72 pg/mg protein, 95% CI -2.66, 1.21, *p*=0.428; did not live in a shelter: coefficient -0.45 pg/mg protein, 95% CI -1.59, 0.70, *p*=0.419). As shown in **Figure 4E**, the change in oxytocin level was not significant irrespective of maternal K6 score (maternal K6 ≤ 4: coefficient -0.56 pg/mg protein, 95% CI -1.88, 0.76, *p*=0.387; maternal K6 ≥ 5: coefficient -0.45 pg/mg protein, 95% CI -2.17, 1.28, *p*=0.560).

### Changes in HRV Measures Among Mothers and Children

The results of the HRV measurements and changes in the HRV measurements by playful mother–child interaction are shown in **Table 3** and **Supplementary Table 1**, respectively. Regarding the changes in mothers following the interaction (**Table 3**), LF and LF/HF were significantly increased by the interaction (LF: coefficient 0.36, 95% CI 0.12, 0.61, *p*=0.005; LF/HF: coefficient 0.12, 95% CI 0.05, 0.19, *p*=0.002). A similar trend was observed irrespective of the sex of the child. Regarding changes in children, HF significantly decreased by 0.29 (95% CI -0.57, -0.01, *p*=0.040) while LF/HF significantly increased by 0.05 (95% CI 0.01, 0.08, *p*=0.008). The decrease in HF was more evident in girls (girls: coefficient -0.34, 95% CI -0.67, -0.01, *p*=0.045; boys; coefficient -0.20, 95% CI -0.84, 0.45, *p*=0.497). Root mean square of successive differences (RMSSD) and standard deviation of normal to normal R-R interval (SDNN) did not change following playful mother–child interaction for both mothers and children.

**Table 3:** Changes in heart rate variability measures among mothers and children.

	All samples	Boys	Girls
	Mean (95%CI)	Mean (95%CI)	Mean (95%CI)
**Changes in mother**	(n=32^a^)	(n=12^a^)	(n=20^a^)
RMSSD	0.16 (-3.39, 3.71)	1.50 (-2.45, 5.45)	-0.65 (-6.07, 4.77)
SDNN	3.46 (-0.69, 7.61)	4.51 (-1.94, 10.96)	2.83 (-3.01, 8.66)
LF^b^	**0.36 (0.12, 0.61)**^**c**^	0.32 (-0.06, 0.71)	**0.38 (0.04, 0.73)**^**c**^
HF^b^	-0.13 (-0.30, 0.05)	-0.17 (-0.51, 0.17)	-0.10 (-0.31, 0.11)
LF/HF^b^	**0.12 (0.05, 0.19)**^**c**^	**0.14 (0.01, 0.27)**^**c**^	**0.10 (0.01, 0.19)**^**c**^
**Changes in child**	(n=25^a^)	(n=8^a^)	(n=17^a^)
RMSSD	0.36 (-7.40, 8.12)	7.75 (-14.81, 30.31)	-3.12 (-9.86, 3.62)
SDNN	2.57 (-2.88, 8.02)	5.36 (-8.78, 19.51)	1.26 (-4.61, 7.13)
LF^b^	-0.01 (-0.26, 0.24)	0.05 (-0.31, 0.40)	-0.04 (-0.39, 0.31)
HF^b^	**-0.29 (-0.57, -0.01)**^**c**^	-0.20 (-0.84, 0.45)	**-0.34 (-0.67, -0.01)**^**c**^
LF/HF^b^	**0.05 (0.01, 0.08)**^**c**^	0.05 (-0.02, 0.11)	**0.05 (0.005, 0.10)**^**c**^

RMSSD, root mean square of successive differences; SDNN, standard deviation of all R-R intervals; LF, low frequencies; HF, high frequencies; LF/HF, low frequencies/high-frequencies ratio.

a analysis was limited to the sample with complete information on the outcome measure.

b measurements were analyzed in the log scale.

c P < 0.05 by fixed effect regression analyses.

### Association Between Maternal Oxytocin Response After Mother–Child Interaction and Behavior Problems 2 Years Later

Finally, we assessed whether change in maternal oxytocin level following playful mother–child interaction could predict later child behavior problems (**Table 4**). We found that the increase of change in maternal oxytocin level by playful mother–child interaction was significantly associated with lower CBCL externalizing problem scores 2 years later (coefficient: -1.56; 95% CI -2.71, -0.41; *p*=0.014), after adjustment for other covariates. The increase in maternal oxytocin level was also associated with lower CBCL total and internalizing problem scores, although not reaching statistical significance.

**Table 4:** Association between maternal change in oxytocin level following mother–child interaction in 2015 and the CBCL score in 2 years in 2017 (n=14^a^).

	Crude		Model 1^b^	
	β^c^	95%CI	β^c^	95%CI
CBCL total score	-1.11	(-2.39, 0.16)	-1.25	(-2.86, 0.36)
CBCL internalizing problem score	-0.82	(-1.87, 0.23)	-0.59	(-1.98, 0.80)
CBCL externalizing problem score	**-1.16**^**d**^	**(-2.15, -0.17)**	**-1.56**^**d**^	**(-2.71, -0.41)**

Bolded values indicate statistical significance at p < 0.05.

a analysis was limited to the sample with complete information on the outcome measure.

b adjusted for mothers' age, sex of the child, APQ, and child traumatic experience due to the earthquake.

c beta value indicated the change in CBCL score in 2017 that was associated with one pg/mg protein increase of change in oxytocin level due to the mother–child interaction in 2015.

d P < 0.05 by linear regression analyses.

## Discussion

To our knowledge, this is the first study that examined oxytocin responses following playful mother–child interaction among survivors of natural disasters. We detected a significant increase in maternal oxytocin level after the interaction, especially among mothers with a first-born boy; however, the maternal oxytocin response was blunted by maternal psychological distress and trauma due to the earthquake. We also found that the mother–child interaction was associated with increased sympathetic activity relative to parasympathetic activity among mothers and children. The increase in maternal oxytocin level was also associated with lower CBCL externalizing problem score 2 years later.

Prior studies found that parental interaction with infants aged 4–6 months old increased oxytocin level in the parents (17), and in mothers who provided high levels of affectionate interaction with their infants, although the increase was not detected in mothers who provided a low level of affectionate interaction (40). In Japan, a first-born boy is often given better treatment compared with his younger siblings, especially in the northeastern region of Japan where Tohoku is located (41, 42). A higher status is also given to the first-born son because he will be the main heir of the family and is responsible for looking after elderly parents (41). Although we did not assess the norm of each family, the level of interaction provided by the mother may vary according to the sex and the birth order of the child, leading to the different maternal oxytocin response levels after the interaction. Future research may need to evaluate the level of affection during the interaction.

We also assessed how maternal psychological distress and trauma from the earthquake could affect oxytocin response following playful mother–child interaction. We found that maternal oxytocin response was blunted among mothers who reported psychological distress. This is consistent with previous studies that showed that the oxytocin system can be disrupted by psychological distress (43, 44). Moreover, a blunted oxytocin response due to trauma was observed even among mothers who did not report psychological distress. Although inconclusive, previous studies have reported that the oxytocin system can be disrupted by past traumatic experience (16, 45). Current findings add to the literature that having either past traumatic experience or psychological distress may have deteriorating effects on the oxytocin system. These results suggest that assessing oxytocin level among disaster survivors along with their psychological distress and trauma could be important for monitoring the effectiveness of parenting programs.

In our analysis, there were no significant changes in child oxytocin level after playful mother–child interaction. Feldman et al. reported that parental interaction with their infants increased the oxytocin level of the infants (17). A prior study reported that oxytocin response following mother–child interaction was blunted in children who experienced neglect and lived in orphanages immediately after birth (46), and among children of depressed mothers (47). In a study with children aged 7–16 with primary DSM-5 anxiety disorders, oxytocin levels did not change significantly after mother–child interaction (27). Since the children in our study were all exposed to the earthquake, the traumatic exposure could be one reason for the blunted oxytocin changes. Furthermore, the blunted response may also be explained by the difference of age range of the children in our study (8.8 years old on average), which was older than those in previous studies [e.g., infants (17, 40), or toddlers/preschoolers (28, 46, 48)]. Children in our study may have felt embarrassed to take part in playful parenting interaction, which may have inhibited the predicted boost to oxytocin level. In addition, the timing of measuring oxytocin level may have been too long for children of this age range in our study and the oxytocin level may have returned to its normal level. Future studies are warranted to examine whether the reason for the blunted oxytocin response is a consequence of traumatic exposure, age, experimental settings, or other factors. Future studies should include a control group of children who were not exposed to the earthquake, assess oxytocin level over several time points, and implement this intervention in a more natural setting such as their own home.

We assessed the effect of playful mother–child interaction on HRV, using mothers and children as a surrogate for psychophysiological effects in the short term (31). Maternal LF and LF/HF were significantly increased by the interactions, while no significant changes were detected in maternal HF. Regarding changes in children, HF was significantly decreased while LF/HF was significantly increased. The decrease in HF was more evident in girls. LF is a marker for a combination of sympathetic and parasympathetic activity, HF for parasympathetic activity, and LF/HF for a combination of sympathetic and parasympathetic activity (31, 49). Thus, the change that was observed among mothers and children can be interpreted as sympathetic activity being activated, relative to parasympathetic activity, by the playful mother–child interaction. While numerous studies have shown that intranasal administration of oxytocin can activate parasympathetic activity in human adults (50, 51), Tracy et al. recently reported that administration of oxytocin may also reduce parasympathetic regulation in people under cognitive stress (52). Our study adds to the literature that playful mother–child interaction accompanied with a natural increase of oxytocin level is associated with changes in the autonomic nervous system among mothers and children traumatized by natural disasters. Future studies may be needed to examine whether oxytocin itself or other factors could mediate the effect of the interaction on the changes in HRV that were observed in our study.

We also examined the effect of maternal oxytocin changes before and after the playful interaction on the onset of child behavior problems using CBCL score 2 years later. The analysis revealed that the increase in maternal oxytocin level associated with the interaction was significantly associated with lower CBCL externalizing problem scores 2 years later, after adjustment for other covariates. To interpret the findings, it is possible that the increase of maternal oxytocin level associated with the interaction may have increased the quality of mother–child interaction in daily life. Other studies have also suggested that oxytocin administration to mothers may improve parenting, especially responding to infant behavior cues (53). An alternative interpretation is that the change of maternal oxytocin level associated with the interaction can be interpreted as a proxy measurement of the functional level of the pre-existing oxytocin system that was determined by oxytocin-related genes. Previous studies have found that maternal oxytocin level may be determined by CD38 and oxytocin receptor gene polymorphisms (e.g., OXTR rs53576), which are associated with child behavior problems (54, 55). Nonetheless, we confirmed that only 10 min of playful mother–child interaction increased maternal oxytocin level, which was associated with a decrease in child externalizing problem scores. Our result provides rationale for future larger-scale studies to evaluate the effect of an intervention using playful mother–child interaction to prevent child behavior problems.

To the best of our knowledge, no previous studies have examined the impact of secretion of oxytocin by playful mother–child interaction on later child behavior problems. Since stimulating natural secretion of oxytocin by playful mother–child interaction would be more accessible and economical than intranasal administration of oxytocin, our results suggest a potential preventive intervention for child behavior problems after natural disaster. Further studies are warranted to investigate whether the effect of stimulating natural secretion of oxytocin by playful mother–child interaction varies by frequency and duration of intervention; the severity, duration, and frequency of the trauma; and by the sex of the parents and children in the session.

Our study has several limitations. First, the mothers and children who participated in the study were not a representative sample of the area that was affected by the earthquake, and we could not follow-up all the participants. Mother–child dyads who were severely affected by the disaster may not have participated because they were not able to, while mother–child dyads who experienced minor disruption and damage may not have been interested in participating. Second, because the CBCL was filled out by parents, child behavior problems that were unknown to parents were not be captured (e.g., behavior problems at school). Third, measuring oxytocin level 5 min after the interaction may have been too long for children in our study. The oxytocin level may have returned to its baseline level. Future studies are needed to assess oxytocin level over several time points in both mothers and children. Such studies should also include a control group of children who were not exposed to the earthquake. Fourth, we were not able to assess the effect of father–child interaction. Fifth, sample size for the analysis that assessed the association between maternal oxytocin changes and child behavior problems was small (n=14). Finally, we did not assess parenting style and oxytocin level before the earthquake, thus the response to playful mother–child interaction may be determined by parenting style or oxytocin levels before the earthquake. Nonetheless, the results that are presented in this study shed light on the prevention of child behavior problems after natural disaster.

In conclusion, we found that playful mother–child interaction increased oxytocin level in mothers but not in children among disaster survivors. We also found that maternal oxytocin response could vary depending on maternal and child characteristics. To clarify the reason for the blunted oxytocin response in children in this study, future studies are needed to assess oxytocin level over several time points in both mothers and children, and should include a control group. Finally, playful mother–child interaction could affect the autonomic nervous system in mothers and children, and the change in oxytocin was related to CBCL externalizing problem score 2 years later. Our results suggest a potential preventive intervention for child behavior problems through playful mother–child interaction after a natural disaster.

## Data Availability Statement

All datasets generated for this study are included in the article/**Supplementary Material**.

## Ethics Statement

The studies involving human participants were reviewed and approved by the research ethics committees at the National Center for Child Health and Development (584), the Ethics Committee at Tokyo Medical and Dental University (M2016-148). Written informed consent to participate in this study was provided by the participants' legal guardian/next of kin. Written informed consent was obtained from the individual(s), and minor(s)' legal guardian/next of kin, for the publication of any potentially identifiable images or data included in this article.

## Author Contributions

NN contributed analyses and interpretation of the data, and drafted, reviewed and revised the manuscript. KN contributed sample processing and oxytocin measurements, reviewed and revised the manuscript. TF contributed conceptualization and the design of the study, interpretation of the data, and revisions to the draft and final versions of the manuscript. All authors approved the final manuscript as submitted and agree to be accountable for all aspects of the work.

## Funding

This study was supported by a grant from the Ministry of Health, Labour and Welfare (H24-jisedai-shitei-007) and Grant-in-Aid for Scientific Research on Innovative Areas from the Japan Society for the Promotion of Science (19H04879).

## Conflict of Interest

The authors declare that the research was conducted in the absence of any commercial or financial relationships that could be construed as a potential conflict of interest.

## Supplementary Material

Supplementary Table 1 Mean heart rate variability of mothers and children in pre-interaction period and post-interaction period.
