# Supplementary material for: Oxytocin Response Following Playful Mother–Child Interaction in Survivors of the Great East Japan Earthquake
Source: Front Psychiatry. 2020 Jun 3;11:477. doi: 10.3389/fpsyt.2020.00477 (PMC7283446; doi:10.3389/fpsyt.2020.00477)
Supplement: Supplementary Table 1 — Mean heart rate variability of mothers and children in pre-interaction period and post-interaction period. [file Table_1.docx]

Supplementary Table 1. Mean heart rate variability of mothers and children in pre-interaction period and post-interaction period

|  | **All samples** | | **Boys** | | **Girls** | |
| --- | --- | --- | --- | --- | --- | --- |
|  | Pre^a^ | Post^b^ | Pre^a^ | Post^b^ | Pre^a^ | Post^b^ |
|  | Mean  (SD) | Mean  (SD) | Mean  (SD) | Mean  (SD) | Mean  (SD) | Mean  (SD) |
| **Mothers** | All  mothers  (n=32^c^) |  | Boys’  mothers  (n=12^c^) |  | Girls’  mothers  (n=20^c^) |  |
| RMSSD | 28.50  (10.78) | 28.66  (10.87) | 25.75  (9.74) | 27.25  (9.30) | 30.15  (11.27) | 29.50  (11.87) |
| SDNN | 38.94  (10.59) | 42.40  (13.71) | 37.78  (9.38) | 42.29  (15.40) | 39.64  (11.43) | 42.46  (13.01) |
| LF^d^ | 4.78  (0.74) | 5.14  (0.85) | 4.93  (0.79) | 5.25  (0.87) | 4.69  (0.71) | 5.07  (0.85) |
| HF^d^ | 4.89  (0.76) | 4.77  (0.95) | 4.88  (0.71) | 4.71  (0.97) | 4.90  (0.80) | 4.80  (0.96) |
| LF/HF^d^ | 0.99  (0.13) | 1.10  (0.20) | 1.01  (0.09) | 1.15  (0.24) | 0.97  (0.15) | 1.08  (0.17) |
|  |  |  |  |  |  |  |
| **Children** | All children  (n=25^c^) |  | Boys  (n=8^c^) |  | Girls  (n=17^c^) |  |
| RMSSD | 46.80  (15.17) | 47.16  (21.05) | 45.38  (19.06) | 53.13  (30.41) | 47.47  (13.59) | 44.35  (15.30) |
| SDNN | 57.22  (16.03) | 59.79  (18.92) | 56.45  (22.86) | 61.81  (25.46) | 57.58  (12.50) | 58.84  (15.82) |
| LF^d^ | 6.11  (0.83) | 6.11  (0.83) | 6.00  (1.09) | 6.04  (1.16) | 6.17  (0.71) | 6.14  (0.67) |
| HF^d^ | 6.15  (0.72) | 5.86  (0.79) | 6.25  (0.83) | 6.06  (1.18) | 6.10  (0.69) | 5.77  (0.55) |
| LF/HF^d^ | 0.99  (0.07) | 1.04  (0.09) | 0.95  (0.06) | 1.00  (0.07) | 1.01  (0.07) | 1.07  (0.09) |
| RMSSD, root mean square of successive differences; SDNN, standard deviation of all R-R intervals; LF, low frequencies; HF, high frequencies; LF/HF, low frequencies/high-frequencies ratio | | | | | | |

^a^Pre: Pre-interaction

^b^Post: Post-interaction

^c^analysis was limited to the sample with complete information on the outcome measure.

^d^measurements were analyzed in the log scale.
